# Target Affinity and Structural Analysis for a Selection of Norovirus Aptamers

**DOI:** 10.3390/ijms22168868

**Published:** 2021-08-18

**Authors:** Katja Schilling-Loeffler, Rachel Rodriguez, Jacquelina Williams-Woods

**Affiliations:** Division of Seafood Science and Technology, United States Food and Drug Administration, Dauphin Island, AL 36528, USA; Katja.Schilling-Loeffler@bfr.bund.de (K.S.-L.); Rachel.rodriguez@fda.hhs.gov (R.R.)

**Keywords:** aptamers, systematic evolution of ligands by exponential enrichment (SELEX), affinity, norovirus, virus like particles (VLPs)

## Abstract

Aptamers, single-stranded oligonucleotides that specifically bind a molecule with high affinity, are used as ligands in analytical and therapeutic applications. For the foodborne pathogen norovirus, multiple aptamers exist but have not been thoroughly characterized. Consequently, there is little research on aptamer-mediated assay development. This study characterized seven previously described norovirus aptamers for target affinity, structure, and potential use in extraction and detection assays. Norovirus-aptamer affinities were determined by filter retention assays using norovirus genotype (G) I.1, GI.7, GII.3, GII.4 New Orleans and GII.4 Sydney virus-like particles. Of the seven aptamers characterized, equilibrium dissociation constants for GI.7, GII.3, GII.4 New Orleans and GII.4 Sydney ranged from 71 ± 38 to 1777 ± 1021 nM. Four aptamers exhibited affinity to norovirus GII.4 strains; three aptamers additionally exhibited affinity toward GII.3 and GI.7. Aptamer affinity towards GI.1 was not observed. Aptamer structure analysis by circular dichroism (CD) spectroscopy showed that six aptamers exhibit B-DNA structure, and one aptamer displays parallel/antiparallel G-quadruplex hybrid structure. CD studies also showed that biotinylated aptamer structures were unchanged from non-biotinylated aptamers. Finally, norovirus aptamer assay feasibility was demonstrated in dot-blot and pull-down assays. This characterization of existing aptamers provides a knowledge base for future aptamer-based norovirus detection and extraction assay development and aptamer modification.

## 1. Introduction

Norovirus is the leading cause of acute gastroenteritis worldwide and the main cause of foodborne illness in the United States (US) [1,2]. Norovirus is a non-enveloped virus with a single-stranded positive-sense RNA genome of approximately 7.6 kb [3,4,5]. The genome contains three open reading frames, with the second open reading frame coding for the major capsid protein (VP1). The icosahedral capsid comprises 180 VP1 units. The VP1 protein consists of the conserved shell region and the diverse protruding (P) domain, which itself contains two subdomains (P1 and P2), with the P2 region being the most genetically variable [6]. The genus norovirus is genetically diverse and divided into 10 genogroups (Gs), where GI, GII, GIV, GVIII, and GIX are human pathogens [7]. These genogroups are divided into more than 50 genotypes based on phylogenetic clustering. Genotypes are further divided into strains, identified according to their first geographical occurrence (e.g., Sydney and New Orleans) [8]. In addition to being genetically diverse, norovirus has a high degree of environmental stability. It can remain infectious after incubation at elevated temperatures (stable for at least 30 min at 60 °C), exposure to low pH, ether solution, and repeated freeze-thaw cycles [9,10,11]. Norovirus also exhibits high resistance to commonly used disinfectants, such as chlorhexidine and certain ethanol- and triclosan-based disinfectants [12]. Norovirus is considered a food safety hazard with an infectious dose as low as 18 particles [13], and it is difficult to detect in instances of low-level contamination in food. The analytical challenge lies not only in cases where the virus levels in food are low but in the variety of food matrices implicated in norovirus outbreaks, which include frozen berries [14,15], leafy greens [16], and molluscan shellfish [17,18]. As viral extraction efficiencies are dependent not only on the method utilized but also on the food matrix [19], there is a need for novel extraction methods to address the matrix diversity. In addition, outside of the food sector, there is a need to develop sensitive, rapid tests for on-site diagnostics that enable the detection of a broad spectrum of norovirus genotypes [20].

Aptamers are oligonucleotides that specifically bind a molecular target with high affinity [21], which suggests they can be utilized in affinity-based diagnostics or pathogen extraction. The use of aptamers in food analytical applications, e.g., for pathogen detection, as diagnostic and extraction tools, emphasizes the usefulness of aptamers and has since been recognized and intensively reviewed [22,23,24]. For norovirus, multiple aptamers have been described [25,26,27,28,29,30]. However, aptamer-based assay development has only been investigated in few cases [31], and norovirus aptamers remain largely uncharacterized. In only two of the five studies published from 2013 to 2018 were aptamer-norovirus equilibrium dissociation constants (*K*_d_s) determined, and only for one genotype in each study [25,29]. The reported aptamer *K*_d_s cannot easily be compared, as the *K*_d_ can vary depending on the method of determination [32,33]. Additionally, previous studies have not investigated the characterization of three-dimensional aptamer structures, making future aptamer modification studies more complicated. Due to the lack of knowledge about target affinity and structure of norovirus aptamers, it is difficult to identify an aptamer or aptamer combination suitable for the development of downstream applications (e.g., assay development) without investing in the labor-intensive aptamer selection and/or aptamer characterization processes.

To further develop norovirus aptamer-based assays, this study was designed to characterize a selection of published aptamers (Table 1).

Here, we studied the affinity for norovirus GI.1, GI.7, GII.3, GII.4 Sydney, and GII.4 New Orleans of seven previously described norovirus aptamers. *K*_d_s for each aptamer-target combination was determined by filter retention assays (FRAs) using norovirus virus-like particles (VLPs). VLPs are noninfectious but are antigenically and morphologically consistent with norovirus virions [4]. For structural analysis, the aptamer panel was analyzed by circular dichroism (CD)-spectroscopy. It had been shown that aptamer-biotinylation could affect their target affinity [34]. Hence, the structure analysis included the measurement of the seven biotinylated aptamers and the native aptamers. Moreover, the assay feasibility of the biotinylated aptamers was determined in an aptamer-mediated dot-blot detecting norovirus VLPs, and in an aptamer-mediated pull-down using paramagnetic particles to extract norovirus from a positive stool sample.

## 2. Results

### 2.1. Aptamer Affinity to Norovirus VLPs

**Figure 1:**
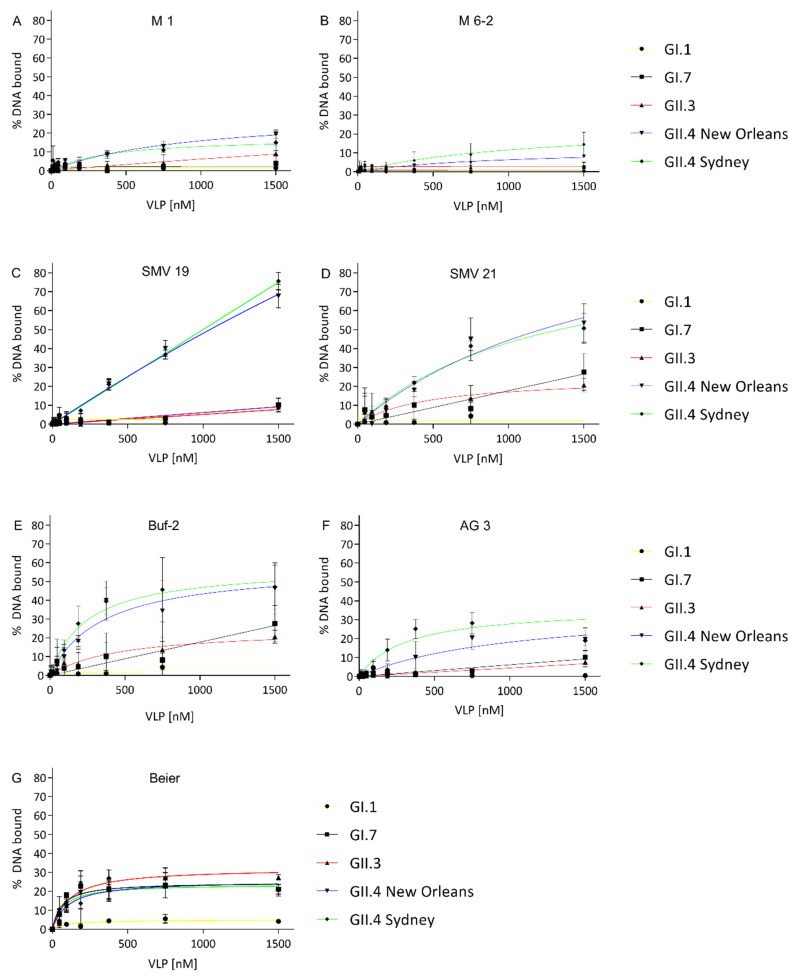
VLP-binding graphs of aptamers M1 (**A**), M6-2 (**B**), SMV19 (**C**), SMV21 (**D**), Buf-2 (**E**), AG3 (**F**) and Beier (**G**). The different curves represent the NoV VLP genotypes GI.1 (yellow), GI.7 (black), GII.3 (red), GII.4 New Orleans (blue), and GII.4 Sydney (green). All graphs are shown with the y-axes indicating bound DNA to target protein from 0 to 80% to allow uniform evaluation of the graphs.

Aptamer-norovirus affinities were determined by FRA. For all aptamers, *K*_d_s were determined when protein-bound DNA exceeded 10% at the highest VLP concentration (1500 nM) tested. Aptamer M1 showed affinity to VLPs of GII.4 New Orleans and GII.4 Sydney (Figure 1A), with *K*_d_s of 963 ± 348 nM and 388 ± 238 nM (Table 2), respectively. M6-2 showed affinity for GII.4 VLPs (Figure 1B) with *K*_d_s in the high nanomolar to a low micromolar range of 928 ± 425 nM and 1130 ± 7895 nM for GII.4 New Orleans and GII.4 Sydney, respectively (Table 2). For SMV19 and GII.4 New Orleans a *K*_d_ in the low micromolar range was determined (Table 2). The SMV19-GII.4 Sydney and -GII.4 New Orleans binding curves exhibited linear character, and no saturation binding was observed (Figure 1C). For SMV21 *K*_d_s of 1777 ± 1021 nM and 1247 ± 372 nM were determined for GII.4 New Orleans and GII.4 Sydney (Table 2), respectively. Additionally, SMV21 exhibited an affinity for GII.3 (Figure 1D) with a *K*_d_ of 464 ± 370 nM (Table 2). Aptamers Buf-2 (Figure 1E) and AG3 (Figure 1F) showed an affinity for GII.4 Sydney with *K*_d_ values of 241 ± 50 nM and 313 ± 81 nM, respectively (Table 2). For VLPs of GII.4 New Orleans, *K*_d_s of 351 ± 89 nM and 1033 ± 433 nM were determined for Buf-2 and AG3, respectively (Table 2). Additionally, Buf-2 showed affinity towards GII.3 with determined *K*_d_ of 465 ± 370 nM (Table 2). The aptamer Beier (Figure 1G) showed a broad binding affinity towards VLPs of the tested norovirus genotypes, except for GI.1 (Figure 1G). These binding curves reached saturation binding at VLP concentrations below 400 nM. This was reflected in the low *K*_d_ values of 63 ± 28 nM, 115 ± 34 nM, 105 ± 47 nM, and 71 ± 38 nM for GI.7, GII.3, GII.4 New Orleans, and GII.4 Sydney, respectively (Table 2).

### 2.2. Structure Analysis of Biotinylated and Non-Biotinylated Oligonucleotides Using Circular Dichroism Spectroscopy

**Figure 2:**
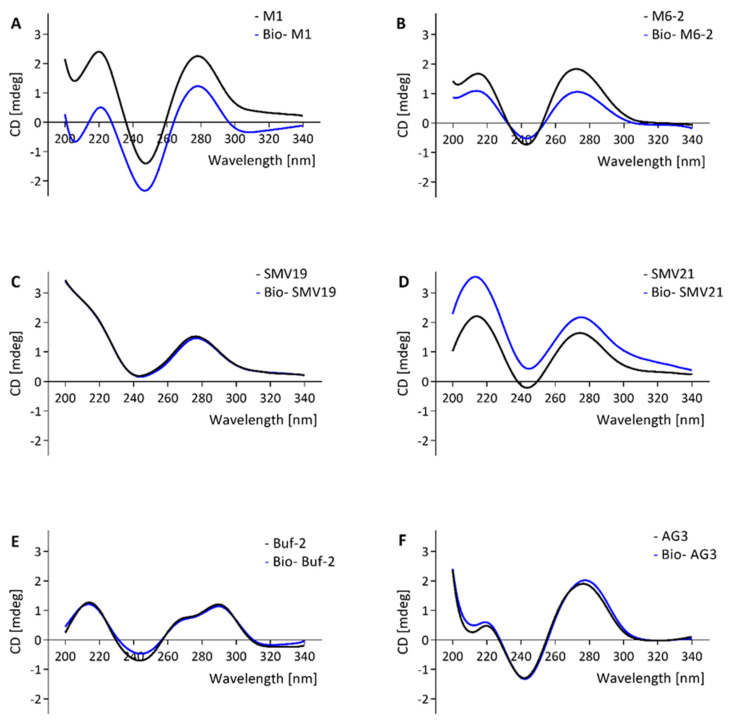

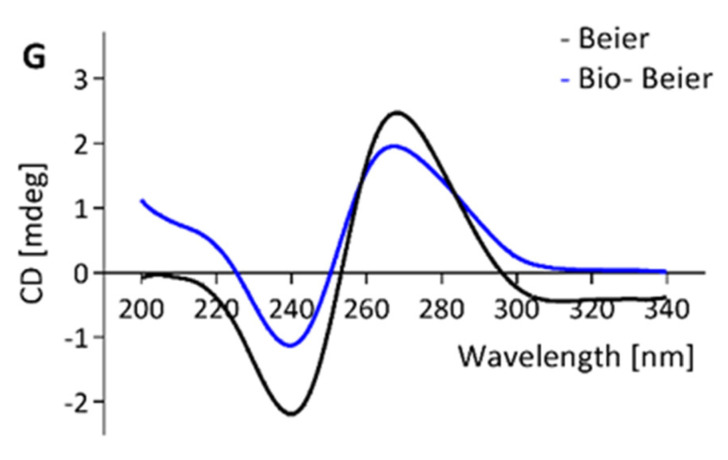
CD spectra of aptamer candidates M1, M6-2, SMV19, SMV21, Buf-2, AG3, and Beier (black curve) and the same biotinylated candidates (blue curve) are shown in (**A**–**G**), respectively.

CD-spectroscopy was used to investigate the molecular structure of the aptamers and the influence of a biotin tag on the molecular folding of the aptamers. Other than Buf-2, the tested aptamers showed a positive band at approximately 270–280 nm and a minimum at approximately 245 nm (Figure 2A–D,F,G). For M1, M6-2, and SMV21, a slight shift in intensities between the spectra for the biotinylated compared to the non-biotinylated aptamer was observed. However, the positive bands at 220 nm and 270 nm, in addition to the negative band at 245 nm, were the same for biotinylated and non-biotinylated aptamers (Figure 1A–D,F,G). Aptamer Buf-2 showed a positive band at 285 nm, a shoulder band at 260 nm, and a negative band at 240 nm (Figure 2E). The CD-spectra obtained for the biotinylated, and non-biotinylated aptamer Buf-2 showed no differences that correlated with the absence or presence of a biotin tag (Figure 2E).

### 2.3. Aptamer-Mediated Dot-Blot for the Detection of Norovirus VLPs

**Figure 3:**
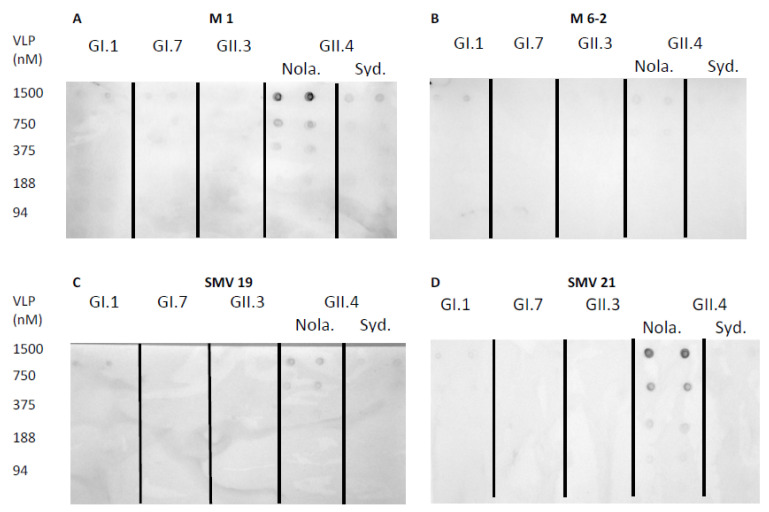

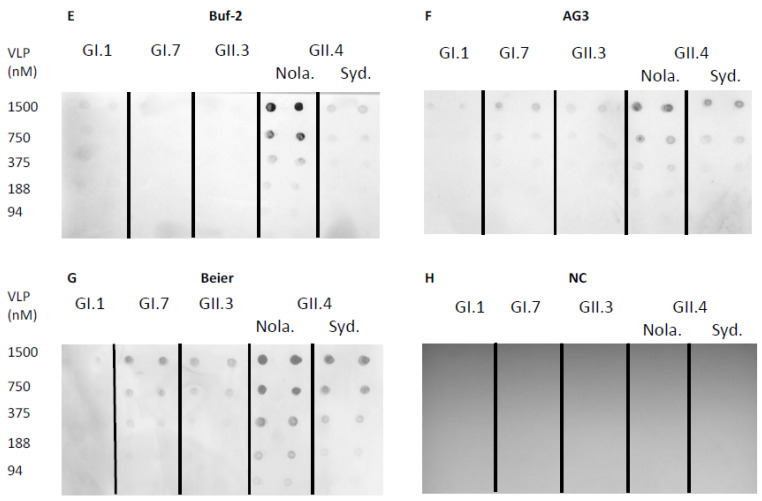
Aptamer mediated dot-blot using selected aptamers and five different genotypes of VLPs. Dot-blot with M1, M6-2, SMV19, SMV21, Buf-2, AG3, and Beier are depicted in (**A**–**G**), respectively. The dot-blot negative control (NC), omitting an aptamer binding step, showed no spots on the membrane (**H**). The concentration of the different VLP solutions applied to the nitrocellulose membrane (0–1500 nM) is indicated on each row’s left side. The different NoV genotypes used are indicated by the abbreviations GI.1, GI.7, GII.3, and GII.4 Sydney (Syd.) and New Orleans (Nola).

Norovirus aptamer utility in a dot-blot assay was determined using norovirus VLPs blotted on a nitrocellulose membrane, which was detected by biotinylated aptamers. Aptamer M1 mediated dot-blot detected GII.4 New Orleans and GII.4 Sydney at concentrations of 375 nM–1500 nM and 1500 nM, respectively (Figure 3A). The aptamer M6-2 mediated dot-blot did not detect the genotypes used in this study at the concentrations tested (Figure 3B). Aptamers SMV19 and SMV21 mediated dot-blots detected GII.4 New Orleans VLPs at concentrations of 750–1500 nM (Figure 3C) and 375–1500 nM (Figure 3D), respectively. In the aptamer-mediated dot-blot using Buf-2 (Figure 3E), GII.4 New Orleans and GII.4 Sydney were detected at concentrations of 188–1500 nM and 750–1500 nM, respectively. Aptamer AG3 mediated dot-blots (Figure 3F) detected GI.7 VLPs at 750–1500 nM and GII.3, GII.4 New Orleans, and GII.4 Sydney VLPs at concentrations of 1500 nM, 375–1500 nM, and 750–1500 nM, respectively. Aptamer Beier mediated dot-blots detected GI.7, GII.3, and GII.4 New Orleans and GII.4 Sydney VLPs in the concentration ranges of 375–1500 nM, 750–1500 nM, 94–1500 nM, and 188–1500 nM, respectively (Figure 3G). No spots were observed on the nitrocellulose membrane for the negative control (Figure 3H).

### 2.4. Aptamer-Mediated Pull-down for the Extraction of Norovirus from Purified Stool Solution

**Figure 4:**
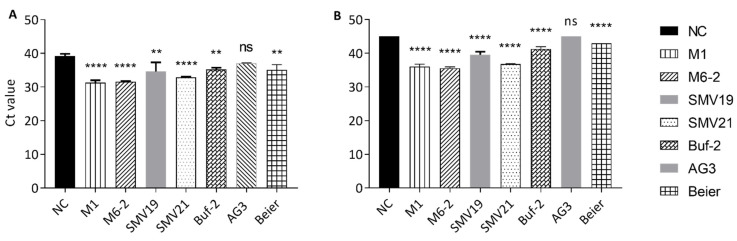
Aptamer-mediated NoV pull-down from stool suspension using different aptamers. The Ct values are shown on the *y*-axis, and each bar on the *x*-axis represents the aptamer used during the pull-down. Statistically significant differences compared to the negative control (NC) are indicated with asterisks above each bar. The *p*-value indication for the asterisks is: not significant (ns) for *p*-value > 0.05, two asterisk (**) for *p*-values between of 0.0021–0.0332 and four asterisks (****) for *p*-values < 0.0001. The aptamer mediated pull-downs were completed for each aptamer in triplicate from a 100-fold dilution from partially purified stool (**A**) and 1000-fold dilution of the same preparation (**B**). In (**B**) no Ct values were obtained for NC and AG3. Therefore, the maximum number of cycles run in the PCR program (45) is shown.

In the aptamer mediated pull-down investigations norovirus GII.4 Den Haag virions were extracted from purified stool solution using biotinylated aptamers and streptavidin-coated paramagnetic beads. In initial studies, nonspecific binding of norovirus to the beads was observed and reduced significantly by blocking with BSA (data not shown). However, the nonspecific binding of the virus to the paramagnetic streptavidin beads could not be entirely eliminated. Therefore, the negative control (blocked beads) was included in the comparative test to investigate whether the aptamer-mediated pull-down resulted in statistically significant improved norovirus extraction compared to the negative control (Figure 4). Pull-downs using M1, M6-2, SMV19, SMV21, and Buf-2 resulted in significantly higher recovery of GII.4 Den Haag compared to the negative control, from both the 100-fold and the 1000-fold dilutions of the purified stool solution (Figure 4). AG3 mediated pull-down recovery of GII.4 Den Haag was not significantly different compared to the negative control (Figure 4) in 100- or 1000-fold dilutions of the purified stool solution. Norovirus was not detected in the AG3 mediated pull-down and the negative control for the 1000-fold dilution (Figure 4B). Beier mediated pull-down resulted in significantly higher GII.4 Den Haag recovery, compared to the negative control, from both dilutions of the purified stool solution (Figure 4).

## 3. Discussion

This study investigated a panel of previously described aptamers (Table 1) for target affinity to norovirus GI.1, GI.7, GII.3, GII.4 Sydney, and GII.4 New Orleans VLPs, for molecular structure and utilization in analytical applications. Aptamers used for diagnostic assays commonly show target affinities with *K*_d_s ranging from picomolar to micromolar range [35]: for the FDA-approved aptamer Pegaptanib, *K*_d_s for interaction with vascular endothelial growth factor (VEGF) range from 49–130 pM [36]; *K*_d_s assessed for the thrombin binding aptamer, and the target protease ranged from 25–200 nM [37]; for the adenosine/ATP aptamer a *K*_d_ of about 6 µM was determined [38]. Compared to these *K*_d_s, the norovirus aptamers’ *K*_d_s described here demonstrate high (*K*_d_s in the nanomolar range) to moderate (*K*_d_s in the micromolar range) binding affinities for the different norovirus strain VLPs. Recently, additional aptamers for the norovirus GII.4 P-particle were described but not considered in this study due to time constraints. These recently selected norovirus aptamers exhibited *K*_d_s in a similar range (148–932 nM) as described for norovirus aptamers’ *K*_d_s determined in this study [30].

The M1 and M6-2 aptamers were selected for norovirus GII.4 P-domain and exhibited affinity towards GII.4 Sydney and GII.4 New Orleans, yet not for GI.1, GI.7, or GII.3. M1 and M6-2 exhibited low overall binding to the norovirus VLPs. Aptamers SMV19 and SMV21 were originally selected using norovirus GII.2 virions with reported *K*_d_s of 191 nM and 101 nM for GII.2 VLPs, respectively [25]. In this study, both aptamers showed high to moderate affinity towards VLPs of two different GII representatives (GII.4 New Orleans and Sydney and GII.3) and no affinity for GI VLPs. All SMV19-VLP binding curves, regardless of the genotype, did not reach a saturation plateau. This generally indicates nonspecific target-binding [39]. The nonspecific binding of SMV19 to other genotypes may be explained by the absence or mutation of the binding site of the genotypes tested here compared to GII.2. The general observation that M1, M6-2, SMV19, and SMV21 show varying affinities to different genotypes confirms previous findings of enzyme-linked aptamer sorbent assays (ELASA) conducted for M1, M6-2, SMV19, and SMV21 [25,27]. The binding studies completed in this work are consistent with the ELASA results, except for SMV19-GI.7. Contrary to the ELASA results, this study observed only marginal affinity (signal from protein-bound aptamer did not exceed 10% at highest VLP concentration) for SMV19 and GI.7. This conflict in comparability among different methods to determine aptamer-target affinity has previously been described [33] and demonstrates that comparing aptamer affinities for one pathogen is best accomplished by using the same assay.

FRA results show that aptamers can have genotype cross-reactive binding properties even if they were selected for only one genotype. Aptamer AG3 was selected using murine norovirus (MNV) [28] but still showed binding affinity toward human norovirus GII.4 (New Orleans and Sydney). Additionally, SMV21 showed binding to GII.4 New Orleans and Sydney and GII.3, despite SMV21 being generated for norovirus GII.2 [25]. Buf-2 also showed a moderate affinity for GII.3 in addition to GII.4 New Orleans and Sydney, although it was generated using the GII.4 New Orleans P-domain [29]. In the study performed by Beier, an aptamer was generated targeting an *E. coli* produced GII.4 VP1 protein, and the aptamer was chosen based on the highest abundance in the twelfth round nucleic acid pool during aptamer selection. Still, an affinity for norovirus was never determined [26]. In this study, Beier showed a broad and high affinity for VLPs of genotypes GI.7, GII.3, and GII.4 New Orleans and Sydney, but did not show an affinity for GI.1, indicating the absence of the aptamer Beiers’ binding motif in norovirus genotype GI.1. The Beier-VP1 binding complex had not previously been characterized, but a simulation predicted the aptamer to bind the S-region and the hinge region between the S-region and P-domain [26]. Even though the norovirus capsid is highly variable among genotypes (especially the P2 region) [6], previous studies have identified antibodies with GI/GII cross-reactive binding properties [40,41,42], confirming the feasibility of generating ligands (antibodies) with cross-reactive epitopes for norovirus GI/GII capsids. Identified epitopes for GI/GII cross-reactive antibodies were located in the S-region and the C-terminal P1 region of the norovirus capsid [40,42]. In the same norovirus surface region, aptamer Beier was predicted to bind. Hence, a cross-reactive binding site for antibodies and aptamers between different norovirus genotypes could be located in the S-region and/or parts of the P1 region. For aptamers, this hypothesis is further supported by the observation that those aptamers selected using the norovirus GII.4 P-domain as SELEX target (Buf-2, M1, and M6-2) showed an affinity for GII representatives only. Although the P-domain is the most accessible domain on the norovirus capsid and therefore an attractive target for aptamer selection (especially with the intention to select an aptamer to use it as an extraction tool), genotype specificity poses a drawback in broad extraction of norovirus genotypes and emerging norovirus genotypes.

FRA experiments were conducted using the buffer composition used during the original aptamers selection to ensure correct aptamer folding [25,26,27,28,29]. The buffers varied in ion salt composition, and pH 6 for the Beier selection buffer differed from the remaining selection buffers, which are pH 7.4. It is known that the norovirus capsid changes adsorption behavior depending on pH in relation to the isoelectric point (pI) of a given virus [43]. The pI values of VLPs of multiple norovirus genotypes have been estimated to be approximately 6 [44]. Therefore, the question remains whether the broad reactivity of the aptamer is due to the electroneutrality of the capsid protein near or at its pI. VLP amounts available for this study did not allow further evaluation of this hypothesis. However, it is questionable whether testing norovirus aptamers selected at pH 7.4 would result in a loss of affinity at pH 6 due to the change of pH value itself, which is known to affect the secondary structure of nucleic acid [45].

Previously, a biotinylated aptamer (including an 11-carbon spacer) showed decreased affinity compared to the same non-biotinylated molecule [34]. However, the CD analysis of norovirus aptamers and their biotinylated counterparts showed no biotin-tag correlated change in the CD-spectra in this study. The intensity shift observed for M1, M6-2, and SMV21 between the spectra of biotinylated and non-biotinylated aptamer could be explained by slight concentration differences in the measurements of the molecules, as the correlation of concentration and band intensity is also used to quantify DNA in CD-spectroscopy [46]. Yet, the absorption bands in the spectra remained unchanged for all aptamers and their matching biotinylated aptamers. Additionally, the FRA and the dot-blot experiments provided comparable results regarding the selectivity and affinity of the aptamers for the norovirus VLP panel. Yet, the FRA assays were completed using non-biotinylated aptamers and the aptamer-mediated dot-blot using biotinylated aptamers. This indicates that for the aptamers tested here, the aptamer-biotinylation (including a 12-carbon spacer as used in this study) is not associated with a change in the secondary structure.

In CD-analysis, a positive band at approximately 220 nm and between 270–280 nm with negative bands at 210 nm and 240 nm in the absorbance spectrum indicates the secondary structure of a regular B-DNA [47]. A parallel G-quadruplex shows a positive band at 210 nm and 260–265 nm with a negative band at 240 nm, whereas an antiparallel quadruplex shows a negative band at 260 nm with a positive band at 290 nm [48]. Contrary to the CD-spectra of M1, M6-2, SMV19, SMV21, AG3, and Beier, the Buf-2 spectra are not consistent with the B-DNA CD-spectrum and only partially consistent with the parallel and antiparallel G-quadruplex CD-spectra. The sequence of Buf-2 does contain a 20 nucleotide stretch with high guanine (G) abundance, including four successive sections of a G triplicate (5′ GGGTTCGGGTTTGGGTTGGG-3′). This could result in the inter- or intramolecular formation of a G-quadruplex. The Buf-2 CD-spectrum shows high resemblance with a telomeric region’s CD spectrum, which can fold into a potassium-induced parallel/antiparallel G-quadruplex hybrid, exhibiting the same positive band around 290 nm, the shoulder band around 265 nm, and negative band at 240 nm [49]. Therefore, it can be suggested that the Buf-2 molecular structure resembles a parallel/antiparallel G-quadruplex hybrid. The truncation of Buf-2 by eliminating the 3′- and 5′-end of the aptamer sequence on either side of the G4-quadruplex core showed that both are involved in P-domain binding (see Supplementary Material Table S2 and Figures S1 and S2).

The aptamer-mediated dot-blot as conducted in this study proved feasible for detecting VLPs with the majority of the aptamers, resulting in detecting VLP concentrations between 188 nM and 1500 nM. The Beier aptamer detected even the lowest VLP concentration used, 94 nM (GII.4 New Orleans). These results are within the range of previously conducted aptamer-mediated dot-blots, e.g., using the DNAzyme-labeled TBA-aptamer in combination with TMB H_2_O_2_ substrate to detect thrombin, where the lowest detected thrombin concentration was 600 nM [50].

The results of the aptamer-mediated pull-down and the dot-blot assays were consistent with the results of the FRA except for the results obtained for AG3 and M6-2. Results from the FRA and dot-blot studies suggest that AG3 shows an affinity for norovirus GII.4 strains. However, AG3-mediated pull-down experiments did not facilitate significantly improved virion extraction compared to the negative control. The affinity of M6-2 to the panel of VLPs as determined in the FRA and the dot-blot assays was low. However, the pull-down assay results showed good virus recovery for both M1 and M6-2 aptamers. The results of the dot-blot using biotinylated aptamers and the results of the FRA using non-biotinylated aptamers were consistent. Additionally, CD analysis of biotinylated and non-biotinylated aptamers did not indicate a change in molecule structure based on the presence of the biotin-tag. Therefore, the increased performance of M1 and M6-2 during the pull-down assay could be attributed to a higher, strain-specific affinity for GII.4 Den Haag (used in the pull-down) over GII.4 Sydney and GII.4 New Orleans (used in FRA and dot-blot) and suggests that differences in the amino acid sequence of the norovirus VP1 protein between the strains could indicate a possible binding motif for M1 and M6-2. Overall, the results of the aptamer-mediated pull-down assays demonstrate the feasibility of the system. However, the use of BSA as a blocking agent should be investigated further, as the negative control (consisting of only paramagnetic beads) still recovered norovirus from the 100-fold diluted purified stool solution. To develop a truly specific virus-concentration method, a mechanism to block the magnetic beads from nonspecific binding is needed.

The lack of knowledge about aptamers’ target affinity and structural properties for noroviruses impedes the development of aptamer-based detection and extraction methods. The use of existing aptamers for assay development saves the laborious and expensive aptamer selection process, which also requires specialized expertise. Therefore, this study was designed to characterize previously published aptamers for norovirus. We focused on affinity measurements and the utility of biotinylated aptamers and employed CD-spectroscopy to analyze the aptamers’ secondary structure. As a result, this study provides *K*_d_s for seven norovirus aptamers and five norovirus strains. The data demonstrate that aptamers can have genogroup cross-reactive properties, as was the case for the aptamer Beier, which had not been previously examined. Additionally, we identified multiple aptamers with specific affinity for only one of the tested norovirus genotypes. To evaluate the specificity of the aptamer-norovirus genotype interaction in more profound detail, future studies should include the use of scrambled aptamers for each aptamer tested as nonspecific binding control, as previously described [34]. The limited amount of VLP available did not allow for further investigation in this regard. Previously uncharacterized aptamer molecule structures were identified as B-DNA, except for Buf-2. The Buf-2 CD-spectra showed a high resemblance with an oligonucleotide with a G-quadruple parallel/antiparallel hybrid structure. For all aptamers, the biotinylation using a 12-carbon spacer did not result in a structural change. The results of the aptamer-mediated dot-blot and pull-down showed that aptamers could be used in extraction and detection applications; the results also showed, that optimization and further research are required to establish aptamers as analytical tools. Such an assay optimization for a foodborne pathogen like norovirus needs to extend beyond the analytical properties (e.g., sensitivity, detection limit) to the assay’s performance in clinical and food matrices. To achieve an assay for food and clinical settings, it is necessary to investigate, whether components of these matrices will impair the aptamer binding properties. Additionally, nonspecific binding of matrix components or the analyte itself to assay reagents, e.g., magnetic beads (as was the case in this study), needs to be identified and eliminated. This could be accomplished by using competitor molecules, which block nonspecific binding sites (such as salmon sperm DNA), which is already used in aptamer binding assays and during aptamer selection [51]. The two feasible analytical platforms, a dot-blot and a pull-down assay described in this study provide the foundation for these future investigations.

## 4. Materials and Methods

### 4.1. Oligonucleotides (Aptamers)

All oligonucleotides were obtained from Integrated DNA Technologies, Inc. (Coralville, IA, USA). The aptamer AG3 was used with primer annealing sites in accordance with the original study, and all other aptamers were ordered without flanking primer annealing sites [25,26,27,28,29]. Prior to each use, aptamers were denatured at 85 °C for 5 min, immediately cooled on ice for 15 min, and equilibrated at room temperature for 20 min.

### 4.2. Norovirus VLPs

Norovirus VLPs of genotypes GI.1, GI.7, GII.3, GII.4 New Orleans, and GII.4 Sydney were kindly provided by Robert Atmar of Baylor College of Medicine (Houston, TX, USA) produced, purified, and characterized as previously described [4].

### 4.3. Filter Retention Assay to Investigate Target Binding of Selected Norovirus Aptamers

For each FRA experiment, the selection buffer of each original aptamer was used for membrane equilibration, binding, and washing steps. The buffer compositions for each aptamer were as previously published (Supplementary Material Table S1) [25,26,27,28,29]. The FRA was conducted as previously described [52,53]. Briefly, nitrocellulose membranes (pore size 0.45 µm; Fisher Scientific, Suwanee, GA, USA) were treated with 0.4 M NaOH for 10 min, washed three times in water, and then equilibrated in selection buffer. Aptamers were labeled with [γ-32P]-ATP (Perkin Elmer, Meridan, CT, USA) using the Promega T4 Polynucleotide Kinase (Promega Corporation, Madison, WI, USA) according to the manufacturer’s instructions. Unincorporated nucleotides were removed using the Zymo Oligo Clean&Concentrator Kit (Zymo Research Corporation, Irvine, CA, USA). Prepared aptamers (final concentration in binding reaction 1 nM) and VLPs (final concentrations in binding reactions 0–1500 nM) were combined, aptamer-VLP binding allowed for 60 min, and 20 µL of the mixture vacuum-filtered through the prepared nitrocellulose membrane and then washed with 600 µL selection buffer. The protein-bound radiolabeled aptamer was detected on the filter by autoradiography using a phosphor storage screen (GE Healthcare, Chicago, IL, USA), and signals were detected with the Typhoon FLA 9000 (GE Healthcare, Chicago, IL, USA) in phosphor imaging mode (50 µm pixel). Autoradiography data were analyzed using the Image Quant software (GE Healthcare, Chicago, IL, USA, 2015–2017), exported to GraphPad Prism 7.02 (GraphPad Software, San Diego, CA, USA, 2015–2017)), and relative binding to the target protein was calculated through a known amount of labeled aptamer, which was blotted directly to the membrane without filtration. *K*_d_ values were determined in triplicate using the one site-specific binding equation, as given below, where T is the target concentration, and Bmax is the concentration of available ligands in saturation binding.

(1) $$\mathrm{Bound}\;\mathrm{Aptamer}=\frac{B_{\max}\left[T\right]}{K_{d}+[T]}$$

### 4.4. Analysis of Oligonucleotides Using Circular Dichroism Spectroscopy

Aptamers and their biotinylated counterparts were diluted in their corresponding selection buffer (supplementary material, Table S1) to achieve a final aptamer concentration of 10 µM. CD spectra were obtained in triplicate in 1 nm steps in the wavelength range of 200 nm to 320 nm using the CD-Spectrometer Jasco 810 (Jasco, Easton, MD, USA) with a 0.1 cm pathway glass cuvette. Three CD scans per aptamer were averaged to create a spectrum, and buffer signals were subtracted. The data were plotted in GraphPad Prism 7.02. To smooth the curves, spectra are shown using the LOWESS function in GraphPad Prism default settings.

### 4.5. Aptamer-Mediated Dot-Blot Detecting Norovirus Virus like Particles

For the dot-blot, duplicates of 2 µL VLP solutions with final concentrations of 0 nM, 94 nM, 188 nM, 375 nM, 750 nM, and 1500 nM were blotted on a nitrocellulose membrane (pore size 0.45 µm; Fisher Scientific, Suwanee, GA, USA). After 15 min, the membranes were submerged in 50 mL phosphate-buffered saline (PBS, Gibco, Fisher Scientific) supplemented with 5% BSA (5%-BSA-selection buffer) and incubated overnight at 4 °C in a closed container. The membrane was washed five times for 5 min each with 40 mL 5%-BSA-selection buffer. For the binding step, 20 mL of 5%-BSA-selection buffer were supplemented with 30 µL aptamer (100 µM) and the preparation was added to the washed membrane. Following 1 h incubation, the membrane was washed three times for 5 min each with 35 mL selection buffer, removed from the buffer, blotted dry on a paper towel, and DNA immediately crosslinked to the membrane using the auto function in the XL-1500 UV Crosslinker (Sepctrolinker, Westbury, NY, USA) for 60 s. Subsequently, the membrane was dried, and aptamers were either immediately detected or stored in a dry environment at approximately 18 °C for no longer than seven days. For the negative control, VLPs were directly blotted on a nitrocellulose membrane (pore size 0.45 µm; Fisher Scientific), and the dot-blot protocol was completed without using aptamers.

For detection, the dried membrane was equilibrated in 40 mL PBS for 15 min. In parallel, 6 µL alkaline-phosphatase conjugated streptavidin (Fisher Scientific) was diluted in 30 mL PBS. The membrane was then placed in streptavidin solution and incubated for 30 min at room temperature, followed by four washing steps with 40 mL PBS for 5 min each, and then blotted dry. All steps were completed at room temperature. The alkaline phosphatase substrate solution was prepared by dissolving an NBT/BCIP Ready-to-Use tablet (Sigma-Aldrich, St. Louis, MO, USA) in 10 mL 18 mΩ water and was added to the membrane. After 40 min incubation in the dark and under horizontal rotation (50 rpm), the reaction was stopped by rinsing the membrane in 18 mΩ water. Under white light, the membrane was photographed using the Gel DOC (Bio-Rad, Hercules CA, USA).

### 4.6. Aptamer-Mediated Pull-Down of Norovirus GII.4 from Purified Stool Solution

For the aptamer-mediated norovirus pull-down, norovirus GII.4 Den Haag was partially purified from a 10% stool suspension in PBS. 15 mL stool suspension was mixed using a vortex, subsequently extracted twice using 5 mL chloroform, and filter sterilized using a low protein-binding filter (Corning Low Protein Binding Filter (0.2 µm), Fisher Scientific). For the norovirus pull-down, the purified stool solution was diluted 100- and 1000-fold in the appropriate selection buffer for the aptamer tested.

For the aptamer-mediated pull-down, 50 µL magnetic beads (Dynabeads My One Streptavidin, Fisher Scientific) were used to recover 200 pmol biotinylated aptamers. An initial study had shown nonspecific virus binding to the streptavidin-paramagnetic beads (data not shown). Therefore, the beads were blocked by washing two times in twice the bead volume using selection buffer supplemented with 1% BSA (1%-BSA-selection buffer), followed by overnight incubation at 4 °C with circular rotation, using setting 4 on a Bellco Roller Drum (Bellco Glass Inc., Vineland, NJ, USA) in twice the bead volume 1%-BSA-selection buffer. The next day, beads were washed two times with twice the bead volume using 1%-BSA-selection buffer. Subsequently, the beads were suspended in one bead volume 1%-BSA-selection buffer and used within 1 h.

For the pull-down, 200 pmol biotinylated aptamer in 50 µL selection buffer was added to 1 mL purified stool solution. After 1 h at room temperature with circular rotation using setting 4 on a Bellco Roller Drum (Bellco Glass, Inc., Vineland, NJ, USA), 50 µL blocked beads were added to the mixture. For the negative control (NC), 50 µL blocked beads were added to the purified stool solution dilutions, without aptamers. After an additional 30 min at room temperature, the beads were collected, and the supernatant discarded. The beads were washed four times with 500 µL 1%-BSA-selection buffer and suspended in 140 µL PBS. The 140 µL bead suspension was extracted using the QIAamp Viral RNA Mini Kit (Qiagen, Valencia, CA, USA) following the manufacturer’s instructions, except for the bead removal. The beads were removed using a magnetic rack after nucleic acid precipitation from virus lysate and before the column-loading step. Extracted norovirus RNA was analyzed by RT-qPCR as previously described [54,55,56]. The pull-down experiments were completed in triplicate. Although the RT-qPCR method used had demonstrated efficiency and reproducibility to quantify virus genomic copy numbers [18], as all samples could be tested in a single run, comparing relative Ct values is more straightforward and appropriate for the relative comparisons made in this study. The average Ct values obtained were compared to the average Ct values of the negative control by the multiple comparisons Dunnett’s test (α = 0.05) following one-way ANOVA, using GraphPad Prism version 7.02 [57]. In the case of no detection in the RT-qPCR, the Ct value was set to the maximum number of PCR cycles run in the experiment, which is 45.

## Figures and Tables

**Table 1:** Summary of selected, previously published norovirus aptamers, their SELEX target, and determined *K*_d_.

Candidate	Sequence 5′-3′	Target Protein	*K*_d_
M1 [27]	TGTTTATGGGGATAAACGTATCTAATTCGTGTACTAATCA	GII.4 P-domain with GST fusion protein	n.d. *
M6-2 [27]	TGGGAAGAGGTCCGGTAAATGCAGGGTCAGCCCGGAGAG		n.d. *
SMV19 [25]	CACCAGTGTGTTGAGGTTTGAGCACACTGATAGAGTGTCA	Whole virus GII.2	191 nM **
SMV21 [25]	CCATGTTTTGTAGGTGTAATAGGTCATGTTAGGGTTTCTG		101 Nm **
Buf-2 [29]	GAAATTGGGTTCGGGTTTGGGTTGGGATTACTTAGCGATG	GII.4 P-domain with His-Tag	17 ± 7 nM
AG3 [28]	GCTAGCGAATTCCGTACGAAGGGCGAATTCCACATTGGGCTGCAGCCCGGGGGATCC	MNV	pM range ***
Beier [26]	GTCTGTAGTAGGGAGGATGGTCCGGGGCCCCGAGACGACGTTATCAGGC	GII.4 VP1 with His-Tag	n.d. *

* n.d.: not determined. ** *K*_d_ was determined using GII.2 VLPs, not the SELEX target. *** no definitive *K*_d_ was published, but *K*_d_ was determined to be in the picomolar range based on binding studies.

**Table 2:** *K*_d_ determined for selected NoV aptamers and VLPs of five different genotypes. When overall binding did not exceed 10%, and a saturation-plateau of the binding curve was not observed, *K*_d_s were not determined as indicated by a dash (-).

Aptamers	*K*_d_ for GI.1 [nM]	*K*_d_ for GI.7 [nM]	*K*_d_ for GII.3 [nM]	*K*_d_ for GII.4 New Orleans [nM]	*K*_d_ for GII.4 Sydney [nM]
M1	-	-	-	963 ± 348	388 ± 238
M6-2	-	-	-	928 ± 425	1130 ± 7895
SMV19	-	-	-	9342 ± 7491	-
SMV21	-	-	464 ± 370	1777 ± 1021	1247 ± 372
Buf-2	-	-	465 ± 370	351 ± 89	241 ± 50
AG3	-	-	-	1033 ± 433	313 ± 81
Beier	-	63 ± 28	115 ± 34	105 ± 47	71 ± 38

## Supplementary Materials

The following are available online at https://www.mdpi.com/article/10.3390/ijms22168868/s1.
