# Supplementary material for: Target Affinity and Structural Analysis for a Selection of Norovirus Aptamers
Source: Int J Mol Sci. 2021 Aug 18;22(16):8868. doi: 10.3390/ijms22168868 (PMC8396345; doi:10.3390/ijms22168868)
Supplement: Supplementary file 1 [file ijms-22-08868-s001.zip › ijms-1316911-supplementary.pdf]

Supplementary Material

1. Original selection buffers for each aptamer

Table S1. SB Buffers used during different aptamer experiments for the corresponding Aptamers.

Aptamer	SB-Buffer Composition
M 1, M 6-2, SMV 19, SMV 21	PBS (Gibco, purchased through Fisher Scientific, Suwanee GA, USA), pH 7.4
Buf-2	2 mM KCl, 1 mM $MgCl_2$, 1 mM $CaCl_2$ 10 mM Tris Base, 100 mM NaCl, pH 7.4
Beier	5 mM KCl, 2 mM $MgCl_2 \cdot 6H_2O$, 1 mM $CaCl_2$, 3 mM KH_2PO_4 17 mM Na_2HPO_4, 100 mM NaCl, pH 6
AG3	5 mM KCl, 5 mM $MgCl_2 \cdot 6H_2O$, 20 mM Tris Base, 50 mM NaCl, pH 7.4

2. Analysis of Buf-2 structure

To investigate the global three-dimensional structure of the oligonucleotide Buf-2, CD spectra were measured of the full length 40 nt oligonucleotide Buf-2, the 20 nt motif without the 3′- end (Buf-2 variant 1), the 20 nt motif without the 5′- end (Buf-2 variant 2), and the 20 nt motif by itself (Buf-2 variant 3) (Table S2). The CD spectra of the different oligonucleotides (Figure 1) showed that each oligonucleotide exhibited a different absorbance pattern in the spectral region of 220 nm to 340 nm.

Table S2. Names and sequences of Buf-2 variants investigated by CD spectroscopy (letters in red show the 5′-end from the G4-region, blue letters show the G4-region, and green letters show the 3′end from the G4-region).

Name	Sequence 5′-3′
**Buf-2**	GAAATTGGGTTCGGGTTTGGGTTGGGATTACTTAGCGATG
**Buf-2 variant 1**	GAAATTGGGTTCGGGTTTGGGTTGGG
**Buf-2 variant 2**	GGGTTCGGGTTTGGGTTGGGATTACTTAGCGATG
**Buf-2 variant 3**	GGGTTCGGGTTTGGGTTGGG

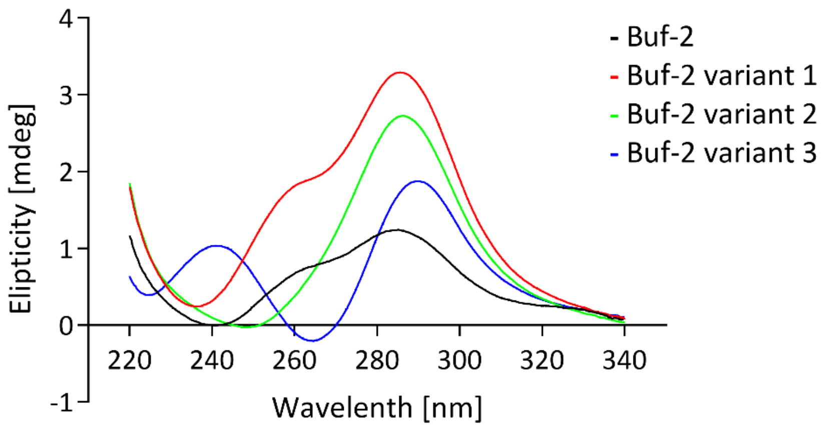

Figure S1. CD spectra of different oligonucleotides derived from aptamer Buf-2 to investigate Buf-2 structure. CD spectra of oligonucleotides were recorded in SB. The curves shown in the spectra were smoothed using the LOWESS function in GraphPad Prism.

Oligonucleotide Buf-2 exhibited two maxima at 288 nm and 265 nm, but no clear minimum between the peaks. For oligonucleotide Buf-2 variant 1, a similar pattern was observed with two maxima at 288 nm and 261 nm without a clear minimum between the peaks. The CD curve of oligonucleotide Buf-2 variant 2 showed only one maximum at 289 nm and a minimum at 252 nm.

The curve of Buf-2 variant 3 showed two distinct maxima at 289 nm and 243 nm and a minimum at 265 nm.

In efforts to determine the P-domain (produced, purified, and characterized as previously described [1]) binding motif and to truncate the 40 nt aptamers, the P-domain affinity of the different Buf-2 variants was investigated via FRA. The detected autoradiography signal for the different oligonucleotides is shown in Figure S2 (A). Studies were conducted in duplicate. Maximum protein binding of oligonucleotide Buf-2 was normalized to 100 %, and P domain binding of the Buf-2 variants was calculated in relation. The binding curves are shown in Figure S2 (B).

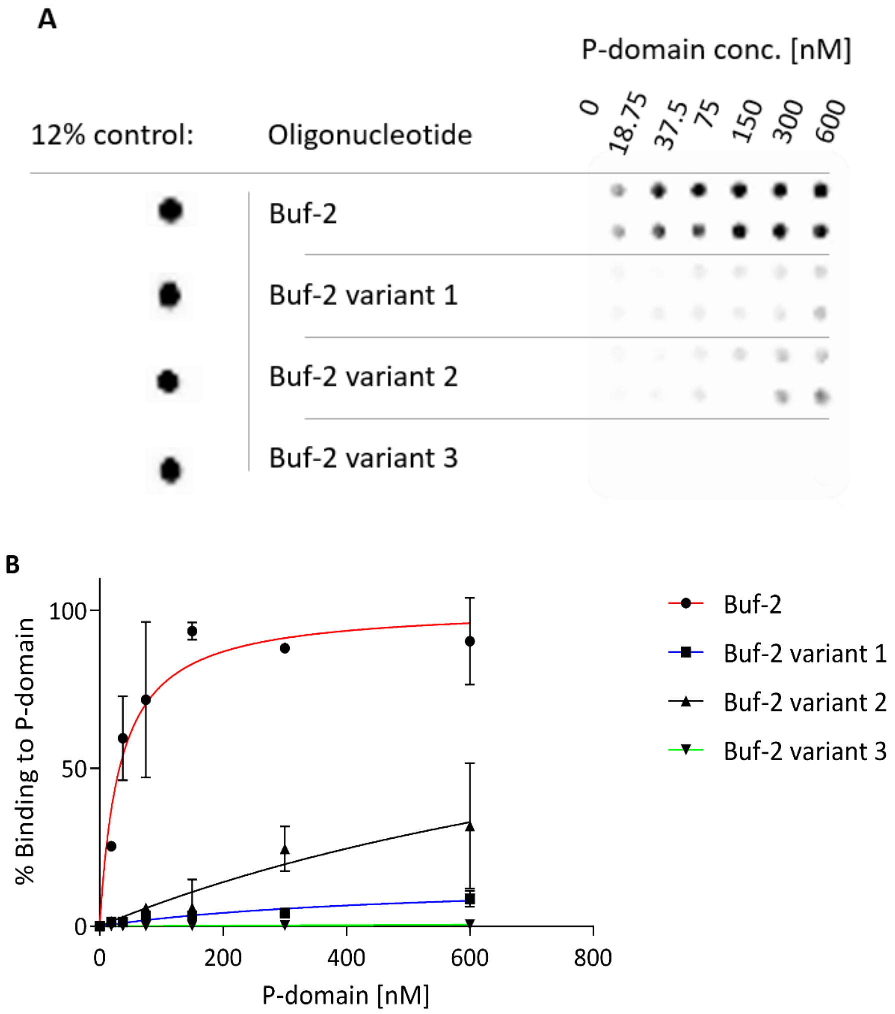

Figure S2. P-domain binding curves of different oligonucleotide variants derived from aptamer Buf-2. The binding of the different Buf-2 variants was tested via FRA. (**A**) Image of autoradiography signals after an FRA. The positive control, equaling 12 % of the labeled oligonucleotide in the filtrate, is shown to the left. FRA was completed in duplicate per variant and for a P-domain concentration range of 0-600 nM. (**B**) The graph shows the matching blotted curves of the FRA.

The FRA studies show that none of the Buf-2 variants exhibited P-domain binding. In conclusion, results of the binding studies assessing the affinity of Buf-2 and the Buf-2 variants for the P-domain revealed that the aptamer Buf-2 could not be truncated without loss of its P-domain affinity

Reference

1. Schilling, K.B.; DeGrasse, J.; Woods, J.W. The influence of food matrices on aptamer selection by SELEX (systematic evolution of ligands by exponential enrichment) targeting the norovirus P-Domain. *Food Chem.* **2018**, *258*, 129.
